# Effectiveness of negative pressure wound therapy (NPWT) in preventing incisional surgical site infection after stoma closure: a single institutional retrospective study

**DOI:** 10.1007/s00595-024-02983-y

**Published:** 2024-12-27

**Authors:** Ryo Nakanishi, Heita Ozawa, Naoyuki Toyota, Ritsuto Akutsu, Shin Fujita

**Affiliations:** Department of Colorectal Surgery, Tochigi Cancer Center, 4-9-13 Yohnan, Utsunomiya, Tochigi 320-0834 Japan

**Keywords:** Surgical site infection, NPWT, Stoma closure

## Abstract

**Purpose:**

Recent findings suggest that utilizing negative pressure wound therapy (NPWT) concurrently with stoma closure may decrease the risk of incisional surgical site infection (iSSI). However, the specific impact of NPWT on iSSI after stoma closure remains unclear. This study investigated the impact of NPWT on SSI after stoma closure.

**Methods:**

Between January, 2010 and December, 2022, 185 patients underwent stoma closure at our hospital. Multivariate analysis was conducted to identify the risk factors for iSSI, using logistic regression analysis. Propensity score matching (PSM) was performed to balance the effect of potential co-factors of stoma closure with and without NPWT, on the incidence of superficial SSIs.

**Results:**

Multivariate analysis identified that the absence of NPWT was an independent risk factor for iSSIs (Odds ratio [OR]: 11.1; 95% confidence interval [CI]: 1.88–64.9; *P* = 0.0078). Following cohort matching, the NPWT-absence and NPWT-presence groups comprised 54 patients each. The incisional SSI rate was significantly lower in the NPWT-presence group than in the NPWT-absence group (0.9%; *n* = 1 vs. 7.4%; *n* = 8, respectively; OR: 9.2; 95% CI 1.11–76.4; *P* = 0.04).

**Conclusion:**

The findings of this study demonstrated that stoma closure with NPWT reduced the SSI rates remarkably. Therefore, NPWT should be considered for stoma closure procedures**.**

## Introduction

The use of a temporarily diverting stoma for minimizing anastomotic complications has become more common when performing low colorectal anastomoses [1]. Stoma closure is a relatively simple procedure; however, incisional surgical site infection (iSSI) is one of its most frequent postoperative complications, resulting in longer hospital stays, more frequent outpatient follow-ups, and higher medical costs [2]. Dermal immersion sutures are used conventionally as skin sutures for primary wound healing during stoma closure, but they are associated with a high rate of wound infection. Circular skin sutures for secondary wound healing have been introduced to reduce the risk of wound infections [3]. Unfortunately, the results of implementing this method in our department have not been satisfactory. Recently, negative pressure wound therapy (NPWT) was introduced to prevent iSSI after gastrointestinal surgery [4, 5]. However, its effect on iSSIs after stoma closure remains unelucidated. We conducted this study to identify the risk factors for iSSI after stoma closure and to clarify the impact of NPWT on stoma closure outcomes.

## Methods

### Patients

The subjects of this retrospective observational study were 185 patients who underwent stoma closure at our institution between January, 2010 and December, 2022. The reasons for and number of temporary stoma cases are as follows: A total of 138 stomas were created intraoperatively for rectal malignancies, including cancer, neuroendocrine tumors, and gastrointestinal stromal tumors; and 47 stomas were created for postoperative complications, including anastomotic leakage and rectovaginal fistula. The stoma was located in the ileum in 162 patients, in the transverse colon in 19 patients, and in the sigmoid colon in four patients. The absence of cancer recurrence was confirmed before stoma closure in patients with preexisting malignant tumors. Patients with renal failure requiring maintenance dialysis or those on steroids that required perioperative steroid replacement were excluded from the analysis.

This study was conducted in compliance with the Ethical Guidelines for Medical Research Involving Human Subjects and approved by the Ethics Committee of the Tochigi Cancer Center (approval number: 24-A016). Comprehensive informed consent for the use of clinical data was obtained from all eligible patients. Informed consent was obtained from the parents and/or legal guardians of patients aged < 20 years old.

### Perioperative and postoperative management

All patients were permitted to eat until the night before surgery and were given an oral rehydration solution the morning of surgery. No mechanical or chemical bowel preparations were performed. Showering and umbilical care were carried out 1 day preoperatively, without shaving or hair removal. Body hair was removed after anesthesia induction, using clippers instead of a razor. Cefmetazole sodium 1 g was administered as a prophylactic antimicrobial 30 min preoperatively and every 6 h postoperatively until postoperative day (POD) 1. Surgery was performed under general or spinal anesthesia. The patient was allowed water on POD1, and then solid oral intake on POD3.

### Surgical technique

At our center, skin suturing was done using dermal embedded sutures until July, 2015, and then using circular skin sutures from August, 2015. NPWT (PICO^®^, Smith & Nephew Wound Management KK) was initiated in combination with circular skin sutures from February, 2017. For dermal embedded sutures, a spindle-shaped skin incision was made with temporary closure of the stoma. Preoperatively, the stoma bowel was not closed temporarily and the skin was incised in an annular fashion, approximately 5 mm from edge of the stoma. The intestinal tract was not resected, and only the anterior wall of the intestinal tract was sutured using a monofilament absorbable thread and silk thread with a serosal muscle layer suture (layer-to-layer anastomosis). A functional end-to-end anastomosis was performed using a stapler if the intestinal tract was resected. Doctors and nurses changed their gloves after the gastrointestinal anastomosis was completed. The skin and fascia were closed using absorbable monofilament sutures. Wound cleaning with high-pressure lavage was performed using 200 mL of saline after fascial closure. Antimicrobial-containing sutures were not utilized for abdominal wall sutures. The skin was sutured with absorbable monofilament thread using circular skin or dermal embedded dermal embedded dermal embedded dermal embedded sutures. The circular skin sutures were secured in a width-like fashion so that the wound was open, with a diameter of approximately 10 mm. In the dermal embedded suture group, the wound was protected with a dressing. In the circular skin suture group, the wound was protected using gauze and a dressing and was kept closed until POD4. It was then opened and the gauze was changed according to the amount of exudate. In the NPWT group, NPWT with PICO^®^ was initiated on POD 1, using PICO^®^ pads, 10 × 20 cm in size, which were changed on POD 4, and the NPWT was completed on POD 7.

### Definition of SSI

Postoperative SSIs were classified according to the Centers for Disease Control and Prevention SSI guidelines [6]. Superficial wound infections were classified as superficial incisional SSIs, whereas intraabdominal abscesses and anastomotic leakages were classified as organ/space SSIs. Positive cultures of wound effusion were classified as positive for wound infection, whereas negative cultures and wound redness were classified as negative for wound infection. Intra-abdominal abscess was defined as an abscess observed on CT and anastomotic leakage was defined as the leakage of contrast medium from the anastomotic site, confirmed by intestinal angiography or from the operative description.

### Statistical analysis

Univariate analysis was performed to compare the iSSI incidence between categories. Student’s t-test was used for normally distributed continuous variables, and the Mann–Whitney test was used for non-normally distributed continuous variables. For categorical variables, the chi-square test or Fisher’s exact test was utilized. Multivariate logistic regression analysis was performed to investigate the risk factors for postoperative iSSI. As this was a retrospective study, we used propensity score matching (PSM) to perform 1:1 matching of the following nine factors in the two groups: sex, age, body mass index (BMI), American Society of Anesthesiologists performance status (ASA-PS), presence of diabetes mellitus, smoking history, stoma location, ostomy period, and construction for anastomotic leakage. The cut-off points for age, BMI, and ostomy period were calculated using the median value. The match tolerance for PSM was set to 0.20 and replacement was not allowed. All statistical tests were two-sided. A *p* value < 0.05 was deemed significant. All statistical analyses were performed using JMP Pro version 16 software (SAS Institute Inc., Cary, NC, USA).

## Results

This study comprised 185 patients who underwent stomatal closure. Table 1 summarizes the patients’ characteristics. The median patient age was 65 (range: 20–86) years and the median BMI was 21.9 (13.4–33.2) kg/m^2^. The stoma was located in the ileum in 162 patients, in the transverse colon in 19 patients, and in the sigmoid colon in four patients. The median duration of ostomy was 139 (13–1184) days. Anastomotic leakage was the background in 47 patients. Circular skin sutures were placed in 128 patients and PICO^®^ was used as NPWT in 105 patients. None of the patients complained of pain, skin lesions, or bleeding that could be attributed to NPWT. Table 2 outlines the incidences of postoperative complications. There were 17 (9.1%) iSSIs and 5 (3.9%) o/sSSIs (Table 2). Three patients (1.6%) suffered postoperative hemorrhage, 12 (6.5%) had bowel obstruction, and 6 (3.2%) had anastomotic leakage (data not shown).

**Table 1:** Characteristics of the patients (n = 185)

Sex	Male	129
	Female	56
Age	Median	65 (20–86)
BMI	Median	21.9 (13.4–33.2)
ASA-PS	1	52
	2	119
	3	14
Diabetes	Yes	22
	NO	163
History of smoking	Yes	62
	NO	123
Location of stoma	Ileum	162
	Sigmoid colon	4
	Transverse colon	19
Ostomy period, median [range], days		139 (13–1184)
Construction due to leakage	Yes	47
	NO	138
Skin sutures	Circular skin sutures	128
	Dermal embedded sutures	57
Anastomosis	Device	39
	Hand sewn	146
NPWT (PICO^®^)	Yes	105
	NO	80

*BMI* body mass index, *ASA* american society of anesthesiologists and *PS* physical status

**Table 2:** Incidence and outcomes of surgical site infections

iSSI	17 (9.1%)
o/sSSI	5 (3.9%)

*iSSI* incisional surgical site infection, and *o/sSSI* organ/space surgical site infection

Table 3 summarizes the univariate and multivariate analyses of the factors affecting iSSI. Multivariate analysis identified non-NPWT as an independent risk factor for iSSI (Odds ratio [OR]: 11.1; 95% confidence interval [CI]: 1.88–64.9; *P* = 0.0078). Furthermore, a significant difference was found between circular skin suturing with NPWT vs. without NPWT (iSSI rate: 1.6% vs. 3.1%; OR: 5.4; 95% CI 1.01–28.8; *P* = 0.04) (Table 4). Before PSM, significant differences in the ASA-PS and stoma location were observed between the NPWT and non-NPWT groups (Table 5). PSM was used to eliminate any bias (Table 4). In the matched cohort, iSSI rates were significantly lower in the NPWT group than in the non-NPWT group (iSSI rate: 0.9% vs. 7.4%; OR: 9.2; 95% CI 1.1–76.4; *P* = 0.04) (Table 6).

**Table 3:** Univariate and multivariate analyses to detect the risk factors for incisional site infection

		Univariate analysis					Multivariate analysis		
		iSSI ( +)		OR	95% CI	*p*	OR	95% CI	*p*
		**(n = 17)**	**%**						
Sex	Male	14	7.6	2.2	0.59–7.8	0.24			
Age > 65 years	Yes	11	6	2.2	0.78–6.27	0.13			
BMI > 22 kg/m^2^	Yes	9	4.9	1.3	0.46–3.44	0.64			
ASA-PS > 1	NO	6	3.2	1.4	0.51–4.13	0.49			
Diabetes	NO	16	8.7	2.3	0.28–18.1	0.43			
History of smoking	NO	6	3.2	1.1	0.32–2.61	0.87			
Anastomosis	Device	4	2.2	1.2	0.35–3.81	0.79			
Location ileum	NO	5	2.7	3.5	1.09–11	0.03	2.1	0.58–7.28	0.26
Ostomy period (day) > 139	Yes	10	5.4	1.5	0.54–4.12	0.43			
Construction due to leakage	NO	14	7.6	1.6	0.45–6.04	0.44			
Circular skin sutures	NO	11	6	4.9	1.7–13.9	0.0032	0.93	0.24–3.51	0.91
NPWT (PICO^®^)	NO	15	8.1	11.9	2.63–53.68	0.0013	11.1	1.88–64.9	0.0078

*BMI* body mass index, *ASA* american society of anesthesiologists, *PS* physical status, *OR* Odds ratio, and *CI* confidence interval

**Table 4:** Comparison of incisional surgical site infections between circular skin suturing with vs. without negative pressure wound therapy

	NPWT absence	NPWT presence	OR	95% CI	*p* value
iSSI	4 (3.1%)	2 (1.6%)	5.4	1.01–28.8	0.04
o/s SSI	2 (1.6%)	3 (2.3%)	3.4	0.53–21.8	0.19

*iSSI* incisional surgical site infection, and *o/sSSI* organ/space surgical site infection. *OR* Odds ratio, and *CI* confidence interval

**Table 5:** Association of negative pressure wound therapy with clinical factors after propensity score matching

Variables		Before propensity score matching					After propensity score matching				
		NPWT Absence		NPWT Presence		*p*	NPWT Absence		NPWT Presence		*p*
		**n = 81**	**%**	**n = 104**	**%**		**54**	**%**	**54**	**%**	
**Sex**	Male	53	28.7	76	41.1	0.42	34	31.5	41	38	0.2
	Female	27	14.6	29	15.7		20	18.5	12	12	
**Age > 65**	Yes	36	19.5	51	27.6	0.65	25	23.1	24	22.2	1
	No	44	23.8	54	29.2		29	26.9	30	27.8	
**BMI > 22**	Yes	34	18.4	54	29.2	0.23	23	21.3	22	20.4	1
	No	46	24.9	51	27.6		31	28.7	32	30	
**ASA-PS > 1**	Yes	43	23.2	90	48.7	< 0.0001	38	35.2	39	36.1	1
	NO	37	20	15	8.1		16	14.8	15	13.9	
**Diabetes**	Yes	6	3.2	16	8.7	0.11	6	5.6	2	1.9	0.27
	No	74	40	89	48.1		48	44.4	52	48.1	
**History of smoking**	Yes	52	28.1	71	38.4	0.75	36	33.3	42	38.9	0.28
	No	28	15.1	34	18.4		18	16.7	12	11.1	
**Stoma location**	Ileum	63	34.1	99	53.5	0.0027	50	46.3	49	45.3	1
	not-Ileum	17	9.2	6	3.2		4	3.7	5	4.6	
**Ostomy Period > 139 days**	Yes	38	20.5	54	30	0.65	24	22.2	27	25	0.7
	No	42	22.7	51	27.6		30	27.8	27	25	
**Construction due to leakage**	Yes	22	11.9	25	13.5	0.61	12	11.1	11	10.2	1
	No	58	31.4	80	43.2		42	38.9	43	39.8	

*BMI* body mass index, *ASA* american society of anesthesiologists and *PS* physical status

**Table 6:** Comparison of incisional surgical site infections with vs. without negative pressure wound therapy following propensity score matching

	NPWT absence	NPWT presence	OR	95%CI	*p* value
iSSI	8(7.4%)	1(0.9%)	9.2	1.11–76.4	0.04
o/s SSI	2(1.9%)	2(1.9%)	1	0.14–7.37	1

*iSSI* incisional surgical site infection and *o/sSSI* organ/space surgical site infection. *OR* Odds ratio and *CI* confidence interval

## Discussion

This single-center, retrospective observational study found that NPWT reduced the risk of iSSI after stoma closure. SSI following stoma closure is associated with prolonged hospital stays, more frequent outpatient follow-ups, and higher medical costs [2]. Circular skin sutures have become widely used for reducing the iSSI incidence after stoma closure [3]. In recent years, the use of NPWT for wounds following stoma closure has been reported to decrease the incidence of iSSI [4, 5]. NPWT is a treatment used to promote wound healing by applying negative pressure to a closed wound. NPWT may be suitable for wounds with high levels of exudate, such as after Circular skin suturing of a stoma closure. In Japan, NPWT was covered by insurance for inpatient treatment in 2010, and its usefulness in open wounds during plastic surgery has been reported [7]. Because NPWT is contraindicated for wounds associated with malignant tumors or residual necrotic tissue, it is important to perform NPWT on the wound surface where necrotic tissue has been removed and infection and inflammation have improved, to maximize its effectiveness. In this study, NPWT with PICO^®^ was started on POD1 after hemostasis of the wound after confirming that there was no residual necrotic material. A PubMed search using the keywords “stoma closure,” “surgical site infection,” and “NPWT” for the period between 1950 and 2023 revealed seven studies using PICO^®^, including our report (Table 7) [8–13]. Only seven studies, including ours, examined the impact of PICO^®^ on iSSI after stoma closure (Table 6). The three studies reported to date all show that PICO^®^ did not reduce the risk of iSSI [8, 11, 13]. In this study, the risk of iSSI was 3.5 times higher for colostomies than for ileostomies (Table 3). The reason why PICO^®^ did not affect iSSI in the study by Uchino et al. could be because all stomas in their study were ileostomies [8]. Considering the differences between the study by Carrano et al. and ours, we changed the pads twice a week for additional reimbursement, whereas they did not change the pads for 7 days after stoma closure [11]. The removal of leachate by flushing the wound with a small amount of saline during pad change, and the continuation of NPWT in this condition, may have contributed to the reduction in iSSI.

**Table 7:** Previous reports of incisional surgical site infection after colostomy closure using negative pressure wound therapy (PICO^®^)

No	Author	Year	Number of patients	Type of stoma	Statistical method	Decline in SSI
1	Uchino^8)^	2016	59	Ileostomy	A randomized control study	No
2	Kim^9)^	2020	36	Ileostomy	A randomized control study	Not shown
3	Obeid^10)^	2021	32	Ileostomy, colostomy	A randomized control study	Not shown
4	Carrano FM^11)^	2021	104	Ileostomy, colostomy	A nonrandomized control study	No
5	Kojima^12)^	2021	30	Ileostomy	A randomized control study	Not shown
6	Kang^13)^	2023	34	Ileostomy, colostomy	A randomized control study	No
7	This study	2024	108	Ileostomy, colostomy	Propensity score matching	Yes

The present study had three strengths. First, to our knowledge, this represents the first report to demonstrate that PICO^®^ reduces iSSI risk following stoma closure. Second, our study had a single-center design; therefore, no intercenter bias was observed in terms of surgical quality, perioperative management, or iSSI criteria. Third, PICO^®^ is more compact and lighter than other NPWT devices, which is advantageous from the point of view of its portability and its ability to be used in ambulatory care. There is a report of reduced wound infection after stoma closure using NPWT with instillation and dwellings (NPWTi-d). Although the removal of necrotic material by continuous washing can reduce iSSI, NPWTi-d instruments are large and cumbersome [14].

The limitations of this study included its single-center design and the fact that it was retrospective with a small sample size, which may have limited its statistical power. Second, PSM was used to eliminate the effects of selection bias, although some residual confounding factors may not have been considered because of the retrospective observational design of the study. The longitudinal nature of this study may have introduced a statistical bias between the groups owing to changes in surgical techniques over the study period; however, no significant changes were observed other than using PICO^®^. Third, the possibility that the surgeon may have influenced the iSSI incidence cannot be ruled out as several doctors were involved in stoma closure during the study period.

## Conclusion

Incisional SSIs after stoma closure were well controlled by perioperative management and infection control, as well as by the concurrent use of PICO^®^ as a postoperative NPWT. The combination of PICO^®^ was found to be effective for reducing iSSI in stoma closure.

## Data Availability

All data generated or analyzed during this study are included in this published article.
